# Embracing critical thinking to enhance our practice

**DOI:** 10.1186/s13244-023-01435-4

**Published:** 2023-05-24

**Authors:** Luis Martí-Bonmatí

**Affiliations:** Medical Imaging Department and Biomedical Imaging Research Group, Hospital Universitario y Politécnico La Fe and Health Research Institute, Valencia, Spain

**Keywords:** Critical thinking, Precision medicine, Reproducibility, Accuracy, Causal inference

Miguel de Cervantes, the great Spanish writer, once wrote that those “who read much and walk much, go far and know much" [1]. The same is true in medicine; reading and gathering experience are the main pillars on which one should develop the knowledge of solving clinical problems in the ever-changing field of healthcare. If properly done, these newly acquired skills will continuously enhance our critical thinking strategies with which we try to identify the best possible improvements in the clinical pathway of radiology. As gaps in knowledge are always present, medicine is rooted in consolidated knowledge based on validated scientific studies and clinical experience reproducibility and accuracy [2]. This represents our best approach to evidence-based decisions. Medical knowledge must be well-established before it can be considered as the basis for decision making and patients guidance in daily practice.

The practice of critical thinking helps us understand the disease manifestations and the related processes and actions that might be relevant to prevent, diagnose and treat diseases. To critically appraise the way we perform evidence-based practice, we must combine best quality research with clinical expertise. This link between exploration and practice will allow radiologists and related disciplines to impact the way medicine is practiced.

These concepts are the cornerstones of *Insights into Imaging*, and it is my privilege as editor-in-chief to describe in this editorial how the journal, and each author, can contribute quality through critical thinking, and hence improve the way we practice radiology by re-shaping our understandings.

It is universally recognized that, in medical imaging, strong levels of evidence are needed to assess the results of the different possible actions and to guide decisions (i.e., to demonstrate a sufficient causal relationship between a specific diagnostic criterion and a disease grading, or a given radiological intervention versus another option in a given condition) toward the most effective or safe outcome considering the benefit of patients and value-based healthcare pathways. Consequently, solid levels of evidence are required to assess the results of different possible actions derived from imaging findings. And, in doing so, we continuously generate more data in our diagnostic and therapeutic activities, whether they are processes or outcomes. This new information will then be transformed into new evidence, real world evidence. In this way, the observed relationship between action and outcome generates causality course actions that will improve our understanding of the best clinical pathways, eliminating the many confounding thoughts that we unconsciously carry during the process of learning and implementing our clinical practice.

Socratic inquiry and Skepticism as foundation. Critical thinking can be understood as the process of analyzing and questioning existing and established knowledge with the intention of improving it. Previous knowledge, either eminence- or evidence-based, should continuously be critically reconsidered and reevaluated for the benefit of the patients, as knowledge is always changing in Precision Medicine. In the real world of medical imaging, this critical thinking must be focused on the evaluation of the effectiveness and clinical impact of all those processes in which images are involved, from the acquisition with different modalities to the processing of the data, from the biological correlation of radiomics as an image biomarker to the therapeutic orientation, and finally in image-guided interventional treatments. Developing critical thinking helps to improve any medical discipline by asking ourselves how to establish better and more precise processes based on existing accumulated evidence, how to recognize and control the biases when approaching a clinical problem, and how to adapt the new clinical information in service of the best solutions. Socratic inquiry and a skeptic attitude can be used to consolidate the best knowledge and construct new associations to be more efficient and to approach excellence in our daily work. Critical thinking is therefore necessary to improve both clinical practice and research in radiology, avoiding disruptive uncertainties and wrong assumptions.

These “questioning and solving” skills require learning, practice, and experience [3], but mainly a recognition of the many uncertainties we do have despite the important scientific advances. Precisely, a good example of the importance of critical thinking is its contribution to Precision Medicine through medical imaging data and information. In daily practice, we should ask ourselves why should we accept a reliable diagnostic method that fails 15% of the time, or an appropriate treatment that is not effective in almost 25% of patients? As scientists, we can improve these clinical decisions in the daily practice. Artificial intelligence (AI) solutions integrating different imaging, clinical, molecular, and genetic data as inputs are being implemented as a suitable pathway to solve clinical problems. The design and methodology of these AI algorithms must allow for their explainability and critical thinking evaluation before they are implemented in clinical practice [4].

In summary, critical thinking develops evidence-based knowledge, provides continuous improvements, and avoids spurious technical and clinical misconceptions. Insights into Imaging is dedicated to manuscripts with a clear critical approach, focusing on excellence in clinical practice, evidence-based knowledge and causal reasoning in radiology. Science is based on long-lived critiques and authors are encouraged to systematically identify, analyze, and solve problems by identifying inconsistencies and correcting errors.

To foster this, *Insights into Imaging* welcomes critical thinking papers and will incorporate a new “Critical Relevance Statement” in all their publications, where authors are asked to summarize in one sentence the question they are trying to answer and the improvement they are providing to the issue at hand.

## Data Availability

Not applicable.
